# Molecular Mechanisms of Alzheimer’s Disease III

**DOI:** 10.3390/ijms232415876

**Published:** 2022-12-14

**Authors:** Ian G. Macreadie

**Affiliations:** School of Science, RMIT University, Bundoora, Vic 3083, Australia; ian.macreadie@rmit.edu.au

This Special Issue of *IJMS* is the third in the series: *Molecular Mechanisms of Alzheimer’s Disease*. Understanding the progression of Alzheimer’s Disease (AD), and especially the biomolecules and pathways involved, can assist with the research and development of intervention strategies. There is considerable evidence of the role of the 40–42 amino-acid peptide known as amyloid beta in the development of the disease, and this collection of papers reinforces its involvement. Interestingly, recent FDA-approved medicines targeting AD utilise monoclonal antibodies designed to remove amyloid beta. The timing of the current treatments may be too late to provide full protection; however, they do provide measurable benefits, supporting the reasons for targeting amyloid beta.

The use of models to study the molecular mechanisms of AD is well accepted, and we are now in a phase of refinement in which more sophisticated questions can be devised. For example, the sporadic nature of AD indicates possible roles for other players interacting with amyloid beta to promote the onset and progression of AD. One such agent, aluminium, has been suspected of involvement in AD for a century (see reviews by Tomljenovic [1] and Kawahara and Kato-Negishi [2]. In a cell-based study by Mcdonald et al. [3], aluminium was shown to exhibit enhanced toxicity in the presence of amyloid beta in a yeast model of AD.

Another toxic metal—the environmental pollutant, mercury—was reviewed by Paduraru et al. [4] for its role in neurodegeneration, citing the evidence for its elevated levels in the blood and brains of AD patients. They also reviewed the interactions between mercury and AD in neuronal degeneration, apoptosis, autophagy, oxidative stress, mitochondrial dysfunction, gastrointestinal microflora, infertility and altered gene expression.

The effects of the third metal ion studied in this issue, calcium—a biologically important, essential metal ion—were reviewed by Guan et al. [5]. Evidence was presented that calcium increased levels of amyloid beta, as well as increasing the aggregation of amyloid beta and phosphorylated tau. These phenomena are all associated with increased progression of AD.

Soto-Rojas et al. [6] have comprehensively reviewed AD with considerable emphasis on the dysfunction of the neurovascular unit (NVU), seen as an essential element of AD pathogenesis. Reduced efficacy of the NVU leads to reduced aerobic metabolism and reduced clearance of amyloid beta from serum. Their review suggests there are opportunities for therapeutic interventions in AD by increasing the performance of the NVU.

The study of neuronal robustness can provide insights about protective factors. Pérez-González et al. [7] studied gene-expression characteristics associated with neuronal resilience in aged-Tg2576 cognitive resilient mice. They performed a transcriptomic analysis using the prefrontal cortex of demented and resilient Tg2576 transgenic AD mice and were able to hypothesize that pathways involved in inflammation, amyloid degradation, memory function and neurotransmission may impact cognitive resilience in AD. Intriguingly, they found a reduction in the influx of peripheral immune cells into the brains of cognitive-resilient subjects. Indeed, *CD4* mRNA expression is significantly reduced in Tg2576 mice with cognitive resilience. For further validation of this result, the authors analysed *CD4* expression in human AD samples, including temporal cortex and peripheral blood mononuclear cells (PBMC), and found a negative correlation between *CD4* mRNA levels in the periphery and the score in the Mini-Mental State Exam of AD patients. These findings highlight the importance of understanding the role of the immune system in the development of neurodegenerative diseases and demonstrate the infiltration of CD4^+^ cells in the brain as a key player of cognitive dysfunction in AD.

Magri et al. [8] provided further understanding and development of mouse AD models in the 3xTg-AD mouse, which is widely used in AD studies. The model has been extensively characterized from both anatomical and behavioural perspectives, but it is poorly studied at the transcriptomic level. Magri et al. [8] characterized the whole blood transcriptome of the 3xTg-AD mouse at three and six months of age and evaluated how its gene expression is modulated by transcranial direct current stimulation (tDCS). RNA-seq analysis revealed 183 differentially expressed genes (DEGs) that represent a direct signature of the genetic background of the mouse. Moreover, in the 6-month-old 3xTg-AD mice, they observed a high number of DEGs that could represent good peripheral biomarkers of AD symptomatology onset. Finally, tDCS was associated with gene expression changes in the 3xTg-AD, but not in the control mice. Their study provides an in-depth molecular characterization of the 3xTg-AD mouse and suggests that blood gene expression can be used to identify new biomarkers of AD progression and treatment effects.

In the previous Special Issue, *Molecular Mechanisms of Alzheimer’s Disease II*, Dhakal et al. [9] established yeast technologies to analyse amyloid beta levels in the presence of therapeutic compounds such as simvastatin [9]. Simvastatin is a hypercholesterolemia drug that coincidentally lowers the incidence of AD and Parkinson’s disease by 50%. This was demonstrated by Wolozin et al. [10], who performed an epidemiological analysis on millions of people with health records held by the US Veterans Administration. Simvastatin users exhibited 50% less Alzheimer’s disease and Parkinson’s disease compared to controls and users of other statins. In this issue, Dhakal et al. [11] extended their studies on yeast to examine natural products that lowered levels of amyloid beta. Further, the versatility of the yeast model allowed combinations of compounds to be examined, revealing synergistic effects in some combinations of natural products. One example of a synergistic combination was *trans*-chalcone plus baicalein. 

To resolve some previous questions in Cerebral Amyloid Angiopathy (CAA), Chatterjee et al. [12] investigated plasma Aβ alterations between pre-symptomatic Dutch-type hereditary CAA (D-CAA) mutation carriers and non-carriers. Plasma Aβ1-40 and Aβ1-42 were found to be lower in mutation carriers compared to non-carriers employing the Single Molecule Array (Simoa) platform. They suggest that plasma Aβ may add value to a panel of biomarkers for the diagnosis of pre-symptomatic CAA; however, further validation studies in larger sample sets are required.

The contributions from these new papers enhance our understanding of AD and its potential treatments.
